# Complete genome sequence of *Desulfurivibrio alkaliphilus* strain AHT2^T^, a haloalkaliphilic sulfidogen from Egyptian hypersaline alkaline lakes

**DOI:** 10.1186/s40793-016-0184-4

**Published:** 2016-09-08

**Authors:** Emily Denise Melton, Dimitry Y. Sorokin, Lex Overmars, Olga Chertkov, Alicia Clum, Manoj Pillay, Natalia Ivanova, Nicole Shapiro, Nikos C. Kyrpides, Tanja Woyke, Alla L. Lapidus, Gerard Muyzer

**Affiliations:** 1 Microbial Systems Ecology, Department of Aquatic Microbiology, Institute for Biodiversity and Ecosystem Dynamics, University of Amsterdam, Amsterdam, The Netherlands; 2 Winogradsky Institute of Microbiology, Research Centre of Biotechnology, RAS, Moscow, Russia; 3 Department of Biotechnology, Delft University of Technology, Delft, The Netherlands; 4 Bioscience Division, Department of Energy Joint Genome Institute, Los Alamos National Laboratory, Los Alamos, NM 87545 USA; 5 Joint Genome Institute, Walnut Creek, CA USA; 6 Biological Data Management and Technology Center, Lawrence Berkeley National Laboratory, Berkeley, CA USA; 7 Department of Biological Sciences, Faculty of Science, King Abdulaziz University, Jeddah, Saudi Arabia; 8 Center for Algorithmic Biotechnology, Institute of Translational Biomedicine, St. Petersburg State University, St. Petersburg, Russia

**Keywords:** *Deltaproteobacteria*, Soda lake, Sediment, Sulfur cycle, Sulfur disproportionation

## Abstract

*Desulfurivibrio alkaliphilus* strain AHT2^T^ is a strictly anaerobic sulfidogenic haloalkaliphile isolated from a composite sediment sample of eight hypersaline alkaline lakes in the Wadi al Natrun valley in the Egyptian Libyan Desert. *D. alkaliphilus* AHT2^T^ is Gram-negative and belongs to the family *Desulfobulbaceae* within the *Deltaproteobacteria*. Here we report its genome sequence, which contains a 3.10 Mbp chromosome. *D. alkaliphilus* AHT2^T^ is adapted to survive under highly alkaline and moderately saline conditions and therefore, is relevant to the biotechnology industry and life under extreme conditions. For these reasons, *D. alkaliphilus* AHT2^T^ was sequenced by the DOE Joint Genome Institute as part of the Community Science Program.

## Introduction

Soda lakes are extreme environments with high salinity and highly alkaline pH values. They are formed in arid regions where high rates of evaporation lead to the accumulation of sodium carbonate salts, which are dominant in these distinctive lakes. Soda lakes support an active microbial sulfur cycle, enhanced by the stability of intermediate sulfur species such as thiosulfate and polysulfides and much lower toxicity of sulfide at these elevated pH conditions. Correspondingly, a wide variety of anaerobic haloalkaliphiles active in the reductive sulfur cycle have been isolated from these lakes [1]. Insights into sulfur redox processes will contribute to understanding how haloalkaliphilic organisms survive and thrive under dual extreme conditions. Some metabolic processes within the reductive sulfur cycle are more favorable under alkaline pH conditions than under circumneutral conditions, such as the disproportionation of elemental sulfur [2]. These sulfur redox processes are not only relevant in natural haloalkaline environments, some wastewater and gas desulfurization treatment plants are often operated at high salt concentrations and pH values where haloalkaliphiles play a role in the remediation of the affected areas. Thus, the haloalkaliphile *Desulfurivibrio alkaliphilus* strain AHT2^T^ was sequenced for its relevance to sulfur cycling and the environmental biotechnology sector by the DOE-JGI Community Science Program.

## Organism information

### Classification and features

**Fig. 1:**
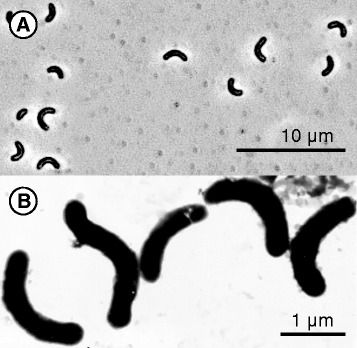
Morphology of *D. alkaliphilus* AHT2^T^. **a** A phase contrast micrograph of the *D. alkaliphilus* AHT2^T^ cells. **b** A scanning electron microscope image of the *D. alkaliphilus* AHT2^T^ cells

**Fig. 2:**
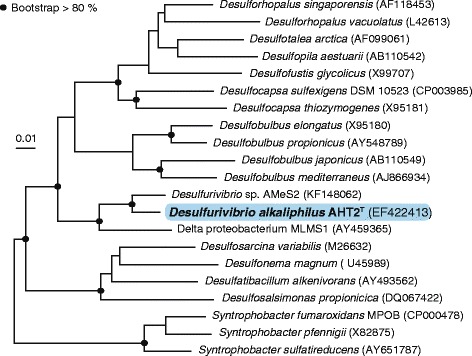
Neighbour joining tree based on 16S rRNA gene sequences showing the phylogenetic position of *D. alkaliphilus* AHT2^T^ to other species within the *Deltaproteobacteria* class. The *Firmicutes* were used as an outgroup and subsequently pruned from the tree. The black dots indicate a bootstrap value between 80 and 100 %. The scale bar indicates a 1 % sequence difference. The tree was constructed with the ARB software package [37] and the SILVA database [19]. The bootstrap values were calculated using MEGA-6 [38]

*D. alkaliphilus* AHT2^T^ is the type strain of the *Desulfurivibrio alkaliphilus* species and was isolated from a mixed sediment sample from eight hypersaline alkaline lakes in the Wadi al Natrun valley in the Libyan Desert (Egypt) [3]. The cells are Gram-negative, non-motile, curved rods that do not form spores (Fig. 1). *D. alkaliphilus* AHT2^T^ tolerates sodium carbonate concentrations ranging from 0.2 - 2.5 M total Na^+^ and grows within a pH range of 8.5 - 10.3 (optimum at pH 9.5) [3]. Phylogenetic analysis showed that the strain belongs to the family *Desulfobulbaceae* within the *Deltaproteobacteria* and is most closely related to a, so far undescribed, haloalkaliphilic chemoautotrophic sulfur-disproportionator within the same genus: *Desulfurivibrio* sp. strain AMeS2 [2]. Strains AMeS2 and AHT2^T^ are, so far, the only known representatives of the *Desulfurivibrio* genus (Fig. 2). The closest sequenced relative to this novel genus, is another soda lake isolate delta proteobacterium sp. MLMS-1, which has been enriched as an arsenate-dependent sulfide oxidizer [4]. *D. alkaliphilus* AHT2^T^ is able to reduce thiosulfate and elemental sulfur [3] and plays a role in the reductive sulfur cycle in soda lake environments [1]. *D. alkaliphilus* AHT2^T^ is also capable of chemolithoautotrophic growth through the disproportionation of elemental sulfur under alkaline pH conditions without iron(III) oxides [2], which are normally required by neutrophilic sulfur disproportionators. More classifications and features are listed in Table 1.

**Table 1:** Classification and general features of *D. alkaliphilus* AHT2^T^

MIGS ID	Property	Term	Evidence code
	Classification	Domain: *Bacteria*	TAS [39]
		Phylum: *Proteobacteria*	TAS [40, 41]
		Class: *Deltaproteobacteria*	TAS [42, 43]
		Order: *Desulfobacterales*	TAS [43, 44]
		Family: *Desulfobulbaceae*	TAS [43, 45]
		Genus: *Desulfurivibrio*	TAS [3, 46]
		Species: *Desulfurivibrio alkaliphilus*	TAS [3, 46]
		Type strain: AHT2^T^	TAS [3]
	Gram stain	negative	
	Cell shape	rod-shaped	
	Motility	non-motile	
	Sporulation	nonsporulating	
	Temperature range	mesophile	
	Optimum temperature	35	
	pH range; Optimum	8.5–10.3; 9.5	TAS [3]
	Carbon source	acetate, HCO_3_^−^	TAS [3]
GS-6	Habitat	hypersaline alkaline lake sediments	
MIGS-6.3	Salinity	moderately salt-tolerant	
MIGS-22	Oxygen requirement	anaerobe	
MIGS-15	Biotic relationship	free living	
MIGS-14	Pathogenicity	none	
MIGS-4	Geographic location	Wadi al Natrun, Libyan Desert (Egypt)	
MIGS-5	Sample collection	September 2000	
MIGS-4.1	Latitude – Longitude	30° 24′ N	
MIGS-4.2		30° 18′ E	
MIGS-4.3	Depth	0–10 cm	TAS [3]
MIGS-4.4	Altitude	−20 m	

## Genome sequencing information

### Genome project history

*D. alkaliphilus* AHT2^T^ was sequenced by the DOE Joint Genome Institute [5] based on its relevance to the biotechnology industry. It is part of the Community Science Program (CSP_788492) entitled ‘Haloalkaliphilic sulfate-, thiosulfate- and sulfur-reducing bacteria’. The project is registered in the Genomes Online Database (Ga0028523) [6] and the complete genome sequence is deposited in GenBank (GCA_000092205). Sequencing and assembly were performed at the DOE Joint Genome Institute using state of the art sequencing technology [7]. A summary of the project information is shown in Table 2.

**Table 2:** Project information

MIGS ID	Property	Term
MIGS-31	Finishing quality	Finished
MIGS-28	Libraries used	Solexa, 454
MIGS-29	Sequencing platforms	454, Illumina
MIGS-31.2	Fold coverage	39.9 × 454, 98 × Illumina
MIGS-30	Assemblers	Newbler,Velvet, phrap
MIGS-32	Gene calling method	Prodigal [17]
	Locus Tag	DaAHT2
	Genbank ID	CP001940
	Genbank Date of Release	01.28.2014
	GOLD ID	Gp0003395
	BIOPROJECT	PRJNA33629
MIGS-13	Project relevance	biotechnological

### Growth conditions and genomic DNA preparation

*D. alkaliphilus* AHT2^T^ was grown anaerobically at 30 °C in Na-carbonate buffered mineral medium containing 0.6 M total Na^+^ with a pH of 10. 4 mM NH_4_Cl, 1 mM MgCl_2_ x 6H_2_O, 1 ml L^−1^ trace element solution [8], 2 mM Na-acetate as C-source and ~5 g/L powdered sulfur (electron acceptor) were added after sterilization. 2 L culture was grown in a 10 L bottle mounted on a magnetic stirrer with an 0.5 bar H_2_ (electron donor) overpressure head-space. The cells from 1 L culture were harvested by centrifugation at 13,000 g for 30 min, washed with 1 M NaCl and stored at −80 °C. The DNA was extracted and purified from frozen pellets by the phenol-chloroform method after pre-treatment with SDS-proteinase K according to Murmur [9]. The purity and molecular weight of the DNA was checked by UV spectroscopy and gel electrophoresis, respectively.

### Genome sequencing and assembly

The total size of the *D. alkaliphilus* AHT2^T^ genome sequence assembly was 3.1 Mbp. The draft genome of *D. alkaliphilus* AHT2^T^ was generated at the DOE Joint Genome Institute using a combination of Illumina [10] and 454 DNA sequencing technologies [11]. An Illumina GAii shotgun library was constructed, which generated 3,998,684 reads and a 454 Titanium standard library, which generated 517,041 reads totalling 123.6 Mb of 454 data. The initial draft assembly contained 57 contigs in 1 scaffold. The 454 Titanium data were assembled with Newbler, 2.0.00.20-PostRelease-11-05-2008-gcc-3.4.6. The Newbler consensus sequences were computationally shredded into 2 kb overlapping fake reads (shreds). Illumina sequencing data was assembled with VELVET, version 1.0.13 [12], and the consensus sequences were computationally shredded into 1.5 kb overlapping fake reads (shreds). We integrated the 454 Newbler consensus shreds and the Illumina VELVET consensus shreds using parallel Phrap, version SPS - 4.24 (High Performance Software, LLC). The software Consed [13] was used in the finishing process as described previously [14]. The final assembly is based on 123.6 Mb of 454 draft data which provides an average 39.9x coverage of the genome and 303.9 Mb of Illumina draft data providing an average 98x coverage of the genome.

### Genome annotation

The complete genome sequence was annotated using the JGI Prokaryotic Automatic Annotation Pipeline [15] with additional manual review using the Integrated Microbial Genomes - Expert Review platform [16]. Genes were predicted using Prodigal [17], followed by a round of manual curation using the JGI GenePRIMP pipeline [18]. Ribosomal RNAs were detected using models built from SILVA [19] and tRNAs were predicted with tRNAScanSE [20]. The predicted coding sequences were translated and used to search the National Center for Biotechnology Information non-redundant database, UniProt, TIGRFam, Pfam, KEGG, COG and InterPro databases. Further annotation was performed using the Integrated Microbial Genomes platform. The final annotated genome is available from the Integrated Microbial Genome system [21].

## Genome properties

The genome is 3,097,763 bp long with GC content of 60.29 % (Table 3). 2732 genes were found, of which 2676 are annotated as protein-coding genes and 56 are RNA genes (47 tRNA genes). A total of 75 % of the protein-coding genes have been assigned a function prediction and 62.26 % have been assigned to a COG (Table 3). The number of genes assigned to each functional COG category is listed in Table 4.

**Table 3:** Nucleotide content and gene count levels of the genome

Attribute	Value	% of total
Genome size (bp)	3,097,763	100.00
DNA coding (bp)	2,806,423	90.60
DNA G + C (bp)	1,867,527	60.29
DNA scaffolds	1	100.00
Total genes	2,732	100.00
Protein coding genes	2,676	97.95
RNA genes	56	2.05
Pseudo genes	56	2.05
Genes in internal clusters	103	3.77
Genes with function prediction	2,049	75
Genes assigned to COGs	1,701	62.26
Genes with Pfam domains	2,280	83.46
Genes with signal peptides	175	6.41
Genes with transmembrane helices	672	24.60
CRISPR repeats	2

**Table 4:** Number of genes associated with general COG functional categories

Code	Value	% of total	Description
J	180	9.50	Translation, ribosomal structure and biogenesis
A	NA		RNA processing and modification
K	72	3.80	Transcription
L	84	4.43	Replication, recombination and repair
B	2	0.11	Chromatin structure and dynamics
D	26	1.37	Cell cycle control, cell division, chromosome partitioning
V	44	2.32	Defense mechanisms
T	134	7.07	Signal transduction mechanisms
M	149	7.86	Cell wall/membrane biogenesis
N	82	4.33	Cell motility
U	50	2.64	Intracellular trafficking and secretion
O	93	4.91	Posttranslational modification, protein turnover, chaperones
C	139	7.34	Energy production and conversion
G	67	3.54	Carbohydrate transport and metabolism
E	129	6.81	Amino acid transport and metabolism
F	53	2.80	Nucleotide transport and metabolism
H	132	6.97	Coenzyme transport and metabolism
I	52	2.74	Lipid transport and metabolism
P	130	6.86	Inorganic ion transport and metabolism
Q	20	1.06	Secondary metabolites biosynthesis, transport and catabolism
R	134	7.07	General function prediction only
S	70	3.69	Function unknown
-	1031	37.74	Not in COGs

## Extended insights from the genome sequence

### Carbon fixation

**Fig. 3:**
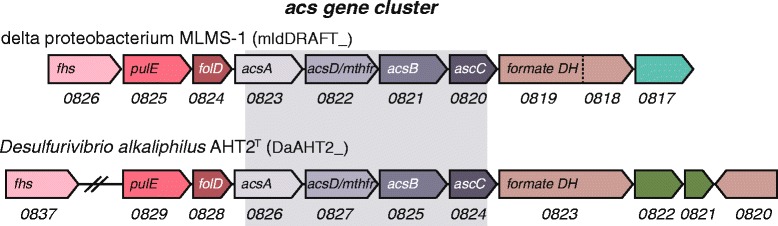
*D. alkaliphilus* AHT2^T^ Wood-Ljungdahl pathway genes, including the *acs* gene cluster, based on delta proteobacterium sp. MLMS-1 [23]. The gene locus tags are depicted beneath the illustrated gene representations

In order to grow chemolithoautotrophically, *D. alkaliphilus* AHT2^T^ assimilates inorganic carbon from the environment. The genome of *D. alkaliphilus* AHT2^T^ contains the key genes necessary for the WL pathway, a mode of carbon fixation from CO_2_, which can run in the reductive and oxidative direction [22]. In the reductive direction, carbon is fixed from inorganic CO_2_ to cell material. The WL pathway functions in this direction in many representatives of sulfate-reducing bacteria within the *Deltaproteobacteria*. Some organisms may couple the reverse, or oxidative, direction to sulfate reduction. The WL gene clusters have previously been defined for delta proteobacterium sp. MLMS-1 from Mono Lake [23], the closest sequenced relative of *D. alkaliphilus* AHT2^T^ (Fig. 2). Here we identified the WL genes necessary for carbon fixation by comparing the corresponding delta proteobacterium sp. MLMS-1 gene clusters to those present in *D. alkaliphilus* AHT2^T^ using the JGI IMG database (Fig. 3). The first step in the reductive pathway is the reduction of CO_2_ to formate, by formate dehydrogenase (DaAHT2_0823 and an accessory protein DaAHT2_0820). This is followed by formyl-THF synthetase (DaAHT2_0837) and a methylene-THF dehydrogenase/cyclohydrolase (DaAHT2_0828) and a methylene-THF reductase (DaAHT2_0827). The *acs* gene cluster is necessary for the carbonyl branch of the reaction [22], which starts with the reduction of CO_2_ to carbon monoxide by a carbon monoxide dehydrogenase (DaAHT2_0826). In the last step, the products of the carbonyl and methyl branch are combined to form the product acetyl-CoA, by a CO dehydrogenase/acetyl-CoA synthase complex (DaAHT2_0825 and DaAHT2_0824). The end product of the WL cycle is typically acetate, however, the genes needed to convert acetyl-CoA to the end product acetate are absent in the *D. alkaliphilus* AHT2^T^ genome, resulting in acetyl CoA being the carbon end product which can be incorporated into biomass.

### Sulfur cycle

Culture studies have provided evidence that *D. alkaliphilus* AHT2^T^ is able to reduce a number of different sulfur redox species to conserve energy [4]. The *dsr* cluster catalyzes sulfite reduction to sulfide [24, 25], which is also present in the *D. alkaliphilus* AHT2^T^ genome consisting of *dsrABC* (DaAHT2_0296, DaAHT2_0297, DaAHT2_2041) and *dsrMK(JOP)* (DaAHT2_2298-DaAHT2_2302). *D. alkaliphilus* AHT2^T^ also has genes which may be involved in the oxidative branch of sulfite disproportionation: a sulfate adenylyltransferase *sat* (DaAHT2_0293) and two adenylylsulfate reductase subunits *aprAB* (alpha: DaAHT2_1471 and beta: DaAHT2_1472). In the haloalkaline environment from which *D. alkaliphilus* AHT2^T^ was isolated, intermediate redox species of sulfur such as polysulfides and thiosulfate are abundantly present. The genes for the reduction of elemental sulfur (polysulfides) and thiosulfate (*psr/phs*) are annotated together as a single KEGG ortholog, namely K08352 [26]. However, the *psr* and *phs* genes have been identified individually in different organisms and are responsible for different reactions.

**Fig. 4:**
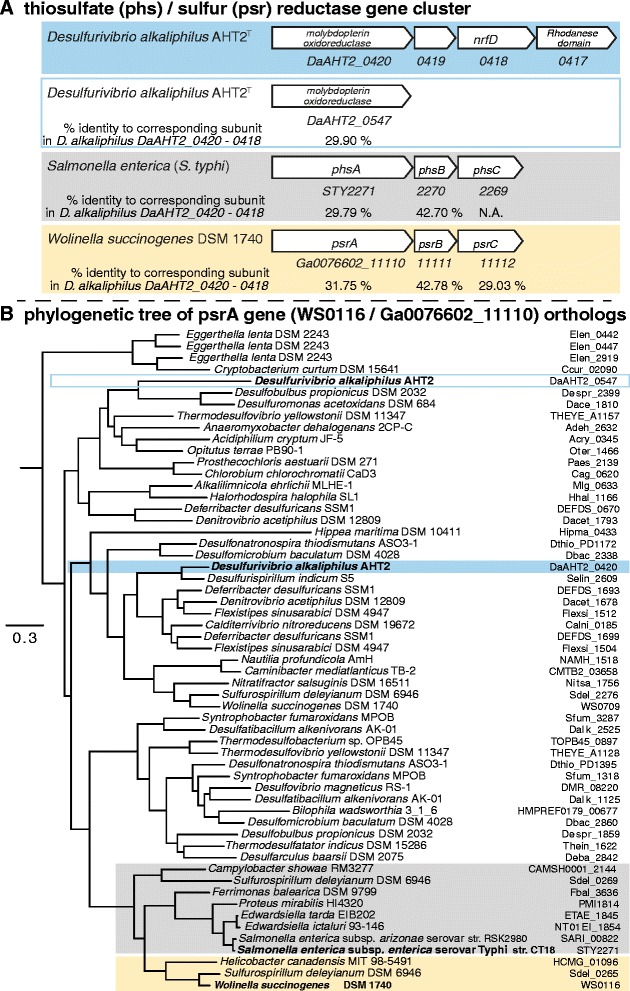
A comparison of the thiosulfate reductase (*phs*) and sulfur reductase (*psr*) gene annotation. **a** The *phs* (grey) and *psr* (yellow) gene clusters and how similar they are to a *phs/psr* gene cluster in *D. alkaliphilus* AHT2^T^ (blue) based on BLAST analysis [47]. **b** A phylogenetic tree of an orthologous group of the *psrA* gene derived from EggNOG (ENOG4107QY8) [31]. Sequences annotated as *phs* are indicated in grey and sequences annotated as *psr* are coloured in yellow. The orthologous genes in *D. alkaliphilus* AHT2^T^ are coloured in blue, and white with a blue outline

The molybdenum-containing polysulfide reductase gene *psrA* (WS0116 / Ga0076602_11110) was first identified in the sulfur/polysulfide-reducing epsilonproteobacterium *Wolinella succinogenes* [27, 28]. The thiosulfate reductase operon *phs* (STY2271-STY2269) was first identified in the enteric bacterium *Salmonella typhimurium* [29, 30]. The genome of *D. alkaliphilus* AHT2^T^ contains two molybdopterin oxidoreductases (DaAHT2_0547 and DaAHT2_0420) (Fig. 4a). In order to determine whether the *D. alkaliphilus* AHT2^T^ gene cluster is a *psr* or a *phs* operon, we used eggNOG 4.5 [31] to find 446 orthologs of *psrA* (WS0116 / Ga0076602_11110) in 233 species, from which a phylogenetic neighbor-joining tree was constructed and trimmed (Fig. 4b). The molybdopterin oxidoreductase sequences of *D. alkaliphilus* AHT2^T^ (DaAHT2_0420 and DaAHT2_0547) did not cluster within the *psr* or *phs* branch (Fig. 4b). Nevertheless, they are part of the same orthologous group as the *W. succinogenes**psrA* (ENOG4107QY8) with which they share 24,80 % (DaAHT2_0547) and 31,75 % (DaAHT2_0420) identity. The *S. typhimurium**phsA* is clustered in the same orthologous group and is 27,34 identical to DaAHT2_0547 and 29,79 % identical to DaAHT2_0420 (Fig. 4a). Only one of the *D. alkaliphilus* AHT2^T^*phsA/psrA* genes is located within an operon of three subunits (Fig. 4a). This means that the *D. alkaliphilus* AHT2^T^ gene with the locus tag DaAHT2_0420 is most probably the active *psrA/phsA*. Laboratory culture evidence points towards the *D. alkaliphilus* AHT2^T^ DaAHT2_4020 – DaAHT2_0418 operon being functional as a sulfur reductase, as it is unable to grow on thiosulfate in absence of H_2_ as electron donor [3]. In addition, the operon is directly adjacent to a sulfur transferase rhodanese domain (DaAHT2_0417), which has been suggested to be essential for the binding, stabilizing and transferring sulfur to the *psrA* subunit [32]. However, more research is needed to define this gene operon as either a *psr* or a *phs* gene cluster.

### Adaptations to the haloalkaline environment

**Fig. 5:**
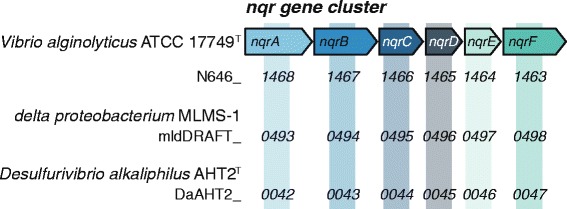
The sodium dependent NADH ubiquinone oxidoreductase (*nqr*) gene cluster. *Vibrio alginolyticus* ATCC 17749^T^ [33, 48] was used as a reference for the delta proteobacterium sp. MLMS-1 and *D. alkaliphilus* AHT2^T^ gene clusters

There are several adaptations that haloalkaliphiles can use to survive in the haloalkaline environment: bioenergetic adaptations, structural membrane adaptations and the use of osmoprotectants to retain osmotic balance [1]. The genome of *D. alkaliphilus* AHT2^T^ contains a voltage gated sodium channel gene *ncbA* (DaAHT2_0077) and the electrogenic sodium/proton antiporter *mrpBCDEFG* operon (DaAHT2_2362 to DaAHT2_2357). The *nqr* operon encodes a sodium pumping NADH: quinone oxidoreductase (alternative to H^+^-pumping conventional NADH-quionone oxidoreductases) that shuttles electrons from NADH to ubiquinone [33, 34]. The *D. alkaliphilus* AHT2^T^ genome contains the first account of the *nqr* operon in anaerobic haloalkaliphiles [35, 36]. The locus tags of the *nqr* gene cluster *nqrA*-*nqrF* in *D. alkaliphilus* AHT2^T^ are DaAHT2_0042 – DaAHT2_0047, and we also found this cluster in *D. alkaliphilus* AHT2^T^’s closest sequenced relative delta proteobacterium sp. MLMS-1 (mldDRAFT_0493-0498) (Fig. 5). The *D. alkaliphilus* AHT2^T^ genome does not contain genes for the synthesis of ectoine or betaine, which function as common osmoprotectants in haloalkaliphiles, but it does have a choline/betaine transporter (DaAHT2_1056).

## Conclusions

In this manuscript we give a short description of the *D. alkaliphilus* AHT2^T^ genome, which was isolated from hypersaline soda lake sediments in the Libyan Desert in Egypt. Its ability to perform inorganic sulfur disproportionation reactions in laboratory cultures indicates that the necessary gene pathways are present in the genome of this organism. The metabolic pathways of disproportionation are so far poorly understood; therefore, further investigation of the *D. alkaliphilus* AHT2^T^ genome may lead to insights which genes are essential to this metabolism. In addition, a more in depth genome sequence analysis might provide more insights into autotrophic carbon metabolism in haloalkaline environments.

## Abbreviations

*acsA*: Carbon monoxide dehydrogenase
*acsB*: Acetyl-CoA synthase
*acsC*: Corrinoid iron-sulfur protein large subunit
*Formate DH*: Formate dehydrogenase
*fhs*: Formyl-H_4_-folate synthase
*folD*: Formyl-H_4_folate cyclohydrolase/methylene-H_4_folate dehydrogenase
*mthfr/acsD*: Methylene-H_4_folate reductase/corrinoid iron-sulfur protein small subunit fusion
*pulE*: Type II secretory pathway ATPase PulE
THF: Tetrahydrofolate
WL: Wood Ljungdahl
